# A cell-based bioluminescence assay reveals dose-dependent and contextual repression of AP-1-driven gene expression by BACH2

**DOI:** 10.1038/s41598-020-75732-z

**Published:** 2020-11-03

**Authors:** Panagiota Vardaka, Teresa Lozano, Christopher Bot, Jonathan Ellery, Sarah K. Whiteside, Charlotte J. Imianowski, Stuart Farrow, Simon Walker, Hanneke Okkenhaug, Jie Yang, Klaus Okkenhaug, Paula Kuo, Rahul Roychoudhuri

**Affiliations:** 1 Department of Pathology, University of Cambridge, Tennis Court Road, Cambridge, CB2 1QP UK; 2 Laboratory of Lymphocyte Signalling and Development, Babraham Institute, Cambridge, CB22 3AT UK; 3 CRUK Therapeutic Discovery Laboratories, Babraham Research Campus, Cambridge, CB22 3AT UK; 4 Imaging Facility, Babraham Institute, Cambridge, CB22 3AT UK

**Keywords:** Biological techniques, Cancer, Cell biology, Immunology, Molecular biology

## Abstract

Whereas effector CD4^+^ and CD8^+^ T cells promote immune activation and can drive clearance of infections and cancer, CD4^+^ regulatory T (T_reg_) cells suppress their function, contributing to both immune homeostasis and cancer immunosuppression. The transcription factor BACH2 functions as a pervasive regulator of T cell differentiation, promoting development of CD4^+^ T_reg_ cells and suppressing the effector functions of multiple effector T cell (T_eff_) lineages. Here, we report the development of a stable cell-based bioluminescence assay of the transcription factor activity of BACH2. Tetracycline-inducible BACH2 expression resulted in suppression of phorbol 12-myristate 13-acetate (PMA)/ionomycin-driven activation of a luciferase reporter containing BACH2/AP-1 target sequences from the mouse *Ifng* + 18k enhancer. BACH2 expression repressed the luciferase signal in a dose-dependent manner but this activity was abolished at high levels of AP-1 signalling, suggesting contextual regulation of AP-1 driven gene expression by BACH2. Finally, using the reporter assay developed, we find that the histone deacetylase 3 (HDAC3)-selective inhibitor, RGFP966, inhibits BACH2-mediated repression of signal-driven luciferase expression. In addition to enabling mechanistic studies, this cell-based reporter may enable identification of small molecule agonists or antagonists of BACH2 function for drug development.

## Introduction

CD8^+^ and CD4^+^ conventional T (T_conv_) cells drive immune activation and promote clearance of infections and cancer. However, their function can also provoke autoimmune and allergic inflammation. The immune system therefore employs a variety of suppressive mechanisms, known as immunoregulatory mechanisms, which act both intrinsically within T_conv_ cells and extrinsically to restrain excessive T cell activation. Immunoregulatory mechanisms also suppress beneficial anti-tumour T cell responses to drive deleterious immunosuppression in cancer. Important among extrinsic immunoregulatory mechanisms is the activity of CD4^+^ regulatory T (T_reg_) cells which limit T_conv_ cell function and promote immune homeostasis and tumour immunosuppression^1–6^. Immunoregulatory mechanisms are therefore important targets for the development of new therapies aimed at treating inflammatory diseases, disorders of excessive immunopathology and cancer.

Appropriate control of T cell differentiation and function requires that they are able to rapidly regulate their gene‐expression programs in response to extrinsic signals. Such capacity is provided by transcription factors (TFs), which bind to the available repertoire of regulatory DNA elements in distinct lymphocyte subsets to program cell‐type‐specific gene expression^7^. Signal-dependent TFs control the response of specific cell types to extrinsic stimuli. In T cells, basic leucine zipper (bZip) TFs of the activator protein 1 (AP-1) family bind to DNA as heterodimers and contribute to activation of gene expression in response to T cell receptor (TCR) signalling^8^. AP-1 TFs, including Jun (c-Jun, JunD, JunB), Fos (c-Fos, Fosb, Fosl1, Fosl2) and BATF (BATF1, BATF2, BATF3), contain bZip domains enabling them to form heterodimeric complexes at palindromic 12-O-Tetradecanoylphorbol-13-acetate (TPA) response elements (TRE; 5′-TGA(C/G)TCA-3′) within regulatory DNA^9,10^. Upon TCR-signalling, AP-1 complexes translocate to the nucleus where they bind to TRE of genes associated with T_eff_ cell differentiation and function^11^.

BACH2 is a 92 kDa transcriptional repressor of the bZip TF family and is predominantly expressed in lymphocytes^12^. It functions as an important regulator of immune activation and transcriptional repression. BACH2 intrinsically regulates the differentiation and function of multiple conventional T cell lineages and is required for efficient development of T_reg_ cells. Deficiency of BACH2 results in a cell-intrinsic defect in T_reg_ cell differentiation, such that C57BL/6 syngenic mice lacking BACH2 protein expression develop lethal inflammation^13^. In addition, BACH2 promotes tumour immunosuppression in a T_reg_-dependent manner^11^. Genetic deletion of *Bach2* in mice results in increased clearance of subcutaneously syngeneic B16 melanoma tumours. Furthermore, the *BACH2* gene in humans is a prominent risk locus for multiple autoimmune and allergic diseases^12^.

The DNA-binding bZip domain of BACH2 is located at the C-terminus of the protein and is required for its repressive activity. In T cells, BACH2 binds to DNA sequences which embed TRE^14^. Through shared possession of bZip domains, BACH2 and AP-1 competitively bind to the same sites within enhancers^11,15^. It has been proposed that such competitive interactions by BACH2 allow it to repress effector-associated gene expression. IFN-γ, encoded by the *Ifng* gene, is an inflammatory cytokine that contributes to antiviral and anti-tumour immunity and can contribute to inflammation and immunopathology^16^. *Ifng* expression is markedly elevated in mouse *Bach2-*deficient CD4^+^ and CD8^+^ T cells^11,17^. In addition, repression of IFN-γ expression is partially required for BACH2 to promote induced T_reg_ (iT_reg_) cell induction^13^. These results suggest that repression of IFN-γ expression is a critical biological function of BACH2, but whether these results derive from direct transcriptional repression of the *Ifng* gene has not been formally established. Moreover, the immunoregulatory function of BACH2 and its predominantly lymphocyte-specific gene expression profile make it a potential target in development of therapies for autoimmune diseases and cancer.

In this work, we have developed a cell-based assay system to report the transcription factor activity of BACH2, wherein tetracycline-inducible BACH2 expression represses AP-1-driven luciferase activity. Tetracycline-inducible BACH2 expression resulted in suppression of phorbol 12-myristate 13-acetate (PMA)/ionomycin-driven activation of a luciferase reporter containing BACH2/AP-1 target sequences from the mouse *Ifng* + 18k enhancer. BACH2 expression repressed the luciferase signal in a dose-dependent manner but this activity was abolished at high levels of AP-1 signalling, suggesting contextual control of AP-1 driven gene expression by BACH2. In addition to enabling mechanistic studies, we propose that this cell-based reporter will enable identification of small molecule agonists or antagonists of BACH2 function for drug development.

## Results

### Generation of a cell line-based luciferase reporter assay of BACH2 repressor function

**Figure 1:**
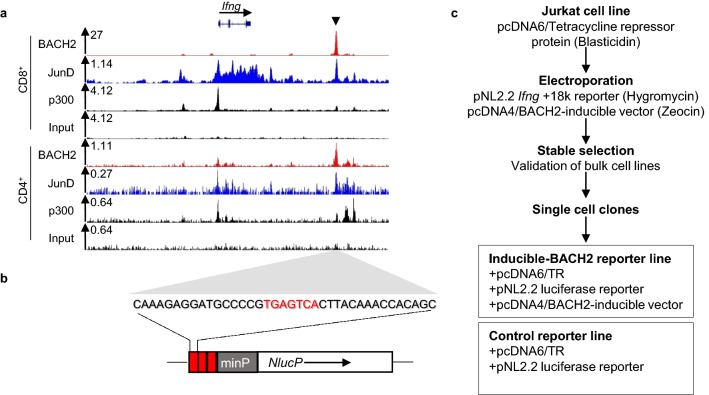
Design and generation of an inducible cell-based luciferase reporter assay for BACH2-mediated repression of AP-1-driven gene expression. (**a**) Analysis of known BACH2, JunD and p300 binding at the mouse *Ifng* locus as determined by ChIP-Sequencing of CD8^+^ and CD4^+^ T cells. A BACH2-bound putative enhancer of *Ifng*, *Ifng* + 18k, is indicated by the black triangle. (**b**) DNA sequence at *Ifng* + 18k containing a TPA response element (TRE; red letters). This sequence was concatenated three times and subcloned upstream of a minimal promoter sequence (minP, grey box) controlling expression of *NlucP* luciferase cDNA sequence in the pNL2.2 reporter vector. (**c**) Experimental schema for generation of clonally derived inducible-BACH2 reporter and control reporter lines. Jurkat cells stably transduced with pcDNA6/TR vector resulting in expression of the tetracycline repressor protein were co-transfected with luciferase reporter (pNL2.2 *Ifng* + 18k) and inducible expression (pcDNA4/BACH2) vectors. Stably transfected cells were selected with hygromycin and zeocin and then subjected to single-cell cloning resulting in the generation of a luciferase reporter line with the potential for inducible BACH2 expression and a control reporter line lacking BACH2-inducibility.

**Figure 2:**
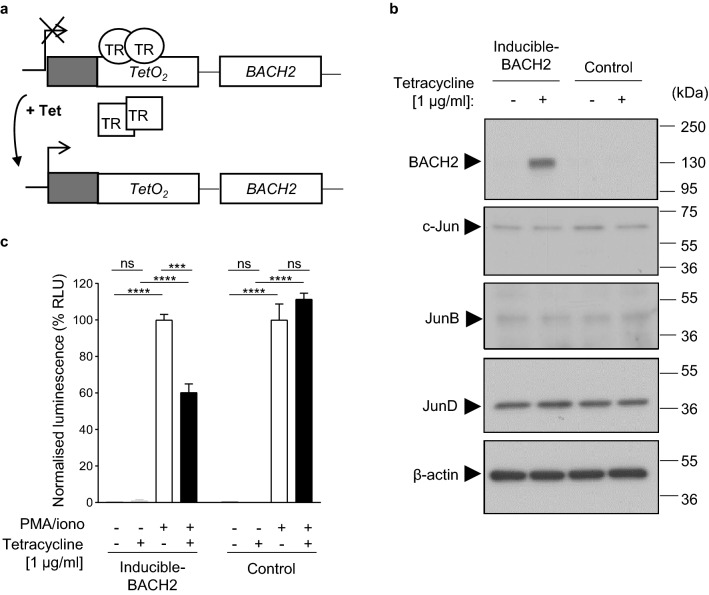
BACH2-mediated repression of AP-1 driven luciferase expression using the inducible reporter system. (**a**) Inducible expression system. Tetracycline repressor (TR) protein in its active form (indicated as a circle) binds to the *TetO*_*2*_ sequence upstream of *BACH2* cDNA subcloned into the pcDNA4/BACH2 vector inhibiting transcription of *BACH2*. Tetracycline (Tet) addition changes the conformation and inactivates the tetracycline repressor (TR) protein (indicated as a square), which is subsequently not able to bind to *TetO*_*2*_ sequence, allowing *BACH2* transcription to commence. (**b**) Western blot for indicated proteins of total lysates isolated from the clonally derived inducible-BACH2 and control reporter cell lines with or without tetracycline treatment. (**c**) Luciferase activity in the inducible-BACH2 and control reporter lines after 6 h PMA/ionomycin stimulation with or without pre-treatment with tetracycline (1 μg/ml). Unpaired two-tailed Student’s *t* test (**c**). Data are representative of 3 independent experiments with 3 culture replicates per condition. Bars and error represent mean (SD); *ns* not significant; ***P < 0.001; ****P < 0.0001.

A putative enhancer of the mouse *Ifng* gene (*Ifng* + 18k), containing a canonical TRE and bound by p300, BACH2, and the AP-1 factor JunD in CD4^+^ and CD8^+^ T cells was identified (Fig. 1a)^11^. A short concatenated DNA sequence surrounding the TRE at *Ifng* + 18 k was subcloned upstream of a minimal promoter (minP) and a luciferase-encoding cDNA sequence (Fig. 1b). We additionally subcloned a human BACH2 cDNA inducible expression vector containing a CMV promoter and control elements from the bacterial tetracycline (Tet) resistance operon. We verified the insert and surrounding vector sequences in both constructed plasmids using Sanger sequencing (Supplementary Fig. 1 and 2). The luciferase reporter and inducible BACH2 expression vectors were co-transfected into Jurkat cells constitutively expressing the Tet repressor protein. Transfected cells were selected using antibiotic selection. Stably transfected single-cell clones were isolated using limiting dilution. A tetracycline-inducible BACH2 functional reporter assay was established in addition to a control reporter lacking inducible BACH2 expression (Fig. 1c). In the developed system, the Tet repressor binds to a specific sequence upstream of *BACH2* cDNA*,* inhibiting BACH2 protein expression. The addition of tetracycline results in a conformational change of the Tet repressor protein, preventing its binding, and allowing *BACH2* to be expressed (Fig. 2a).

We first examined whether BACH2 protein expression is inducible using this system. Cells were treated with or without 1 μg/ml tetracycline, to induce BACH2 expression. Lysates were resolved using sodium dodecyl sulphate polyacrylamide gel electrophoresis (SDS-PAGE) and proteins were detected by western blotting. We observed inducible expression of BACH2 protein upon tetracycline treatment of the inducible-BACH2 reporter line but not of the control reporter line, whereas expression of the AP-1 factors JunB, c-Jun and JunD in total cellular lysates was unchanged (Fig. 2b).

The specificity of anti-BACH2 antibody reactivity was tested by adding a blocking peptide during antibody staining of membranes, which resulted in abolishment of the signal (Supplementary Fig. 3). The inducible-BACH2 reporter line treated with and without tetracycline was stimulated with PMA/ionomycin to cause *Ifng* + 18k enhancer-driven luciferase expression. A ~ 40% reduction of luciferase signal was observed in tetracycline-treated cells, which was not observed in the control reporter line (Fig. 2c). These results indicate that tetracycline-inducible BACH2 protein represses signal-driven luciferase gene expression controlled by a sequence from the consensus *Ifng* + 18k enhancer.

### Dose-dependent repression of AP-1-driven gene expression by BACH2

**Figure 3:**
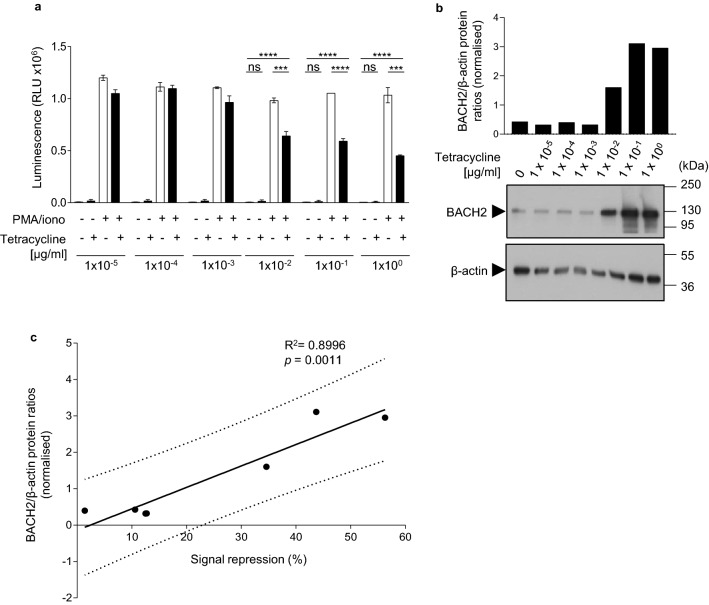
Dose-dependent repression of AP-1-driven gene expression by BACH2. (**a**) Luciferase activity of inducible-BACH2 reporter line after 6 h PMA/ionomycin stimulation with or without pre-treatment with indicated titrated doses of tetracycline. (**b**) Western blot analysis of the abundance of BACH2 protein within total protein lysates from cells in (**a**). Quantified and normalised to β-actin levels of BACH2 protein expression are displayed in the bar graph (top). (**c**) Positive correlation between BACH2 expression normalised to β-actin, and luciferase signal repression at the indicated in (**a**) tetracycline concentrations. Two-way ANOVA with Bonferroni correction (**a**) and linear regression analysis (**c**). Data are representative of 2 independently repeated experiments with 3 culture replicates per condition. Bars and error represent mean (SD); *ns* not significant; ***P < 0.001; ****P < 0.0001.

**Figure 4:**
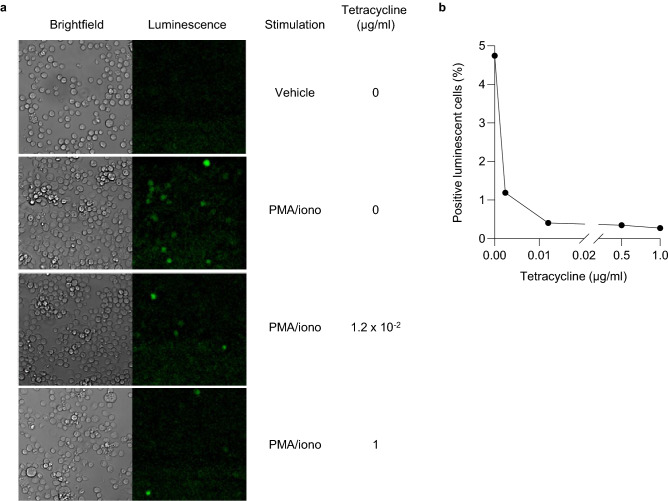
Bioluminescence imaging of BACH2-reporter cells. (**a**) Imaging of inducible-BACH2 reporter cells 6 h after PMA/ionomycin stimulation with or without tetracycline (1 μg/ml) treatment. Unstimulated cells were included (indicated as Vehicle). Images were captured after luciferase substrate addition using brightfield (left) and luminescence (right) channels. Each panel is a representative 300 μm × 300 μm cropped area from the overview image. (**b**) Frequency of positive luminescent cells in stimulated inducible-BACH2 reporter cells following pre-treatment with the indicated doses of tetracycline.

To examine the dose-dependency of BACH2-mediated AP-1-driven signal repression, we performed tetracycline titration experiments. Inducible-BACH2 cells were treated with titrated doses of tetracycline and their luciferase activity was determined following PMA/ionomycin stimulation (Fig. 3a and Supplementary Fig. 4). BACH2 protein expression was also examined by SDS-PAGE and western blotting (Fig. 3b). Luciferase activity was negatively correlated with tetracycline concentration (Supplementary Fig. 5) and a significant positive linear correlation between repression of signal-driven luciferase induction and BACH2 protein expression was observed (Fig. 3c). Imaging of inducible-BACH2 reporter cells after 6 h of PMA/ionomycin stimulation also revealed dose-dependent repression of signal-driven luminescence following treatment of cells with tetracycline (Fig. 4a, b). These data suggest that BACH2 functions as a dose-dependent repressor of AP-1-driven gene expression regulated by sequences derived from the + 18k enhancer of *Ifng.*

### Contextual dose-dependency of AP-1-driven signal repression by BACH2

**Figure 5:**
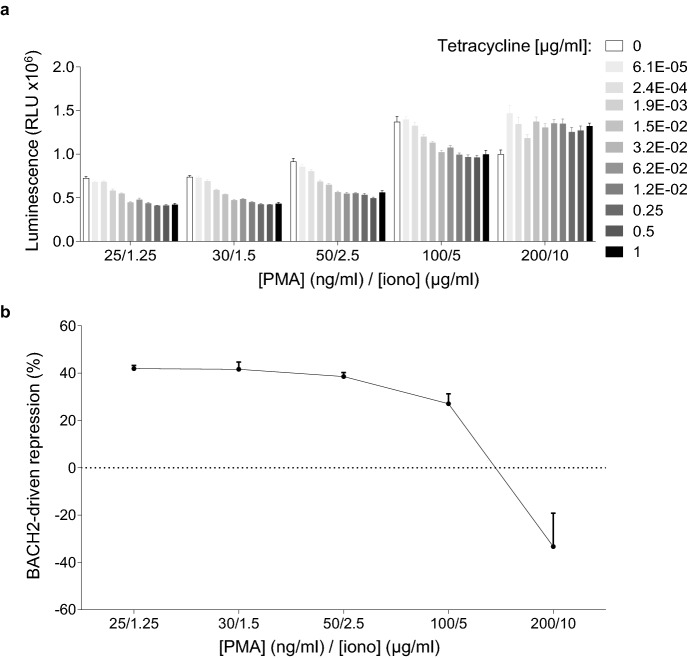
Contextual signal-responsive repression of luciferase expression by BACH2. (**a**) Luciferase activity in inducible-BACH2 reporter line after 6 h stimulation with the indicated concentrations of PMA/ionomycin, with or without pre-treatment with titrated tetracycline doses. Concentrations of tetracycline are indicated in the figure legend. (**b**) Luciferase signal repression at the indicated PMA/ionomycin concentrations with or without tetracycline (1 μg/ml) pre-treatment. (**a**, **b**) Data are representative of 2 independently repeated experiments with 3 culture replicates per condition. Bars and error represent mean (SD).

BACH2 restrains TCR-driven effector differentiation programmes within CD8^+^ T cells^11^. However, despite possessing high levels of BACH2 expression, naïve T cells are able to differentiate into effector cells in the presence of strong levels of TCR stimulation. We asked whether BACH2-mediated repression of AP-1-driven gene expression occurs to the same extent at any level of AP-1 activation or whether its repressor function is limited at saturating levels of AP-1 activation. We therefore stimulated cells with titrated doses of PMA/ionomycin using a single concentration of tetracycline per titration. Importantly, we observed a loss of BACH2-mediated luciferase signal repression at higher levels of PMA/ionomycin stimulation (Fig. 5a, b). These results suggest that BACH2 capacity to mediate AP-1-driven gene expression repression is reduced in the presence of strong activating signals. Thus, dose-dependent AP-1 signal repression by BACH2 is contextual and regulated by the strength of activation signalling in the system.

### BACH2-mediated repression of signal-driven luciferase induction is inhibited by the HDAC3 inhibitor RGFP966

**Figure 6:**
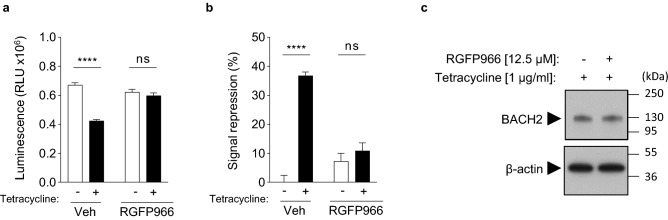
BACH2-mediated repression of luciferase expression is inhibited by the HDAC3 inhibitor molecule RGFP966. (**a**, **b**), Luciferase activity in inducible-BACH2 reporter line after 6 h stimulation with PMA/ionomycin and pre-treatment with or without 1 μg/ml tetracycline and with 12.5 μM of RGFP966 or without (indicated as Veh). (**c**) Western blot analysis of the abundance of BACH2 within total protein lysates from cells in (**a**, **b**) treated with or without 12.5 µM RGFP966. Unpaired two-tailed Student’s *t* test (**a**, **b**). (**a**, **b**) Data are representative of 2 independently repeated experiments with 3 culture replicates per condition. Bars and error represent mean (SD); *ns* not significant; ****P < 0.0001.

There are no known direct activators or inhibitors of BACH2 function. However, it was recently shown that BACH2-mediated repression of the *Prdm1* gene in B cells is partially dependent upon co-recruitment of a complex containing histone deacetylase 3 enzyme (HDAC3). Thus, its repressor function at this locus is inhibited by the HDAC3-selective inhibitor RGFP966^18,19^. We therefore examined whether pre-treatment of cells with RGFP966 would result in inhibition of BACH2-mediated repression in the developed reporter assay. We observed near-complete loss of BACH2-repression of PMA/ionomycin-driven luciferase expression when cells were pre-treated with 12.5 μM RGFP966 (Fig. 6a, b). Importantly, RGFP966 treatment did not affect BACH2 expression in the assay (Fig. 6c). These results provide a positive control for pharmacological inhibition of BACH2 activity in the reporter system developed and shed light on potential mechanisms by which BACH2 represses *Ifng* expression in T cells.

## Discussion

In this study, we have generated a cell-based luciferase reporter assay system to facilitate analysis of the transcriptional repressor function of BACH2 in vitro. Sequences derived from the mouse *Ifng* + 18k enhancer sequence bound by both BACH2 and Jun family AP-1 factors in T cells were used to drive luciferase gene expression. Its signal-driven induction was repressed by tetracycline-inducible BACH2 expression^11^. Our inducible BACH2 reporter system suggests that BACH2-mediated repression of AP-1-driven gene expression is dose-dependent and limited at the highest levels of AP-1 signalling. Previous work has shown that BACH2 represses IFN-γ expression, but whether this was the result of direct control of regulatory elements of the *Ifng* gene had not been tested. These findings suggest that BACH2 represses AP-1-driven induction of *Ifng* through regulatory interactions with AP-1 factors at the *Ifng* + 18k enhancer (Supplementary Fig. 6).

In this study, we examined BACH2-mediated regulation of *Ifng* regulatory elements in a Foxp3-negative conventional T cell line. Repression of IFN-γ expression is a critical biological function of BACH2 not only in conventional CD4^+^ Th1 cells and CD8^+^ T cells, but also during early iT_reg_ cell development, where BACH2-mediated repression of IFN-γ is required for stabilization of iT_reg_ differentiation prior to Foxp3 induction^11–13^. Moreover, we and others have shown that within lineage-committed Foxp3^+^ T_reg_ cells, BACH2 is re-purposed and is not required to maintain Foxp3 expression or suppress IFN-γ expression, but rather blocks the TCR-driven transition between resting T_reg_ (rT_reg_) and activated T_reg_ (aT_reg_) states^20,21^. Given these observations, we chose to study the regulation of *Ifng* expression by BACH2 in the Foxp3-negative Jurkat cell line. It would be useful in future studies, however, to examine the effect of BACH2 on gene regulation at other loci in a T_reg_ cell line, such as the MT-2 human T_reg_ cell line^22^.

Stable transfection of the reporter system allowed for the effect of BACH2 on a chromatinized reporter to be determined, as opposed to commonly utilized transfected plasmid luciferase reporters which are not integrated into the host genome and therefore exist as non-chromatinized plasmid DNA. This system provided the opportunity to examine whether BACH2-mediated repression of gene expression in T cells is in part dependent upon regulation of chromatin. Histone deacetylase 3 enzyme (HDAC3) is found in specific complexes containing NCoR1 and NCoR2 and can be recruited to chromatin by transcriptional repressors^23,24^. In B cells, BACH2 has been shown to interact with NCoR1 and NCoR2 resulting in recruitment of HDAC3 to the *Prdm1* gene. As a result, repression of *Prdm1* by BACH2 is dependent upon the activity of HDAC3^18^. Consistent with these findings, we observed that repression of signal-driven luciferase expression by BACH2 was inhibited upon pre-treatment of cells with the HDAC3-specific inhibitor RGFP966. These findings provide an important positive control for inhibition of BACH2-mediated repressor activity in the developed assay system, relevant to development and validation of high-throughput screening assays. It will also be important in future studies to test the extent to which BACH2-mediated repression of *Ifng* expression in primary cells requires the histone deacetylase function of HDAC3.

A functional relationship between BACH2 and AP-1 factors underlies T cell memory formation^11,12^. BACH2 inhibits both effector and terminal effector differentiation programmes under conditions of weak TCR-signalling, contributing to differentiation of memory CD8^+^ T cells and long-lived responses following viral infection^11^. In our assays, loss of BACH2-mediated repression at high levels of stimulation suggests that BACH2-mediated repression is itself regulated by the strength of activating signals that cells receive. This is consistent with a requirement for T cells expressing high levels of BACH2 to nevertheless be able to differentiate into effector cells in the presence of strong TCR and inflammatory signalling. Indeed, a number of regulatory pathways are known to affect the post-translational stability, localisation and function of BACH2 and an opportunity to further interrogate their role in a reductionist system is provided by this assay. However, such investigations would need to be complemented by corresponding assays using more physiological systems, including in primary T cells.

Finally, this cell-based reporter provides an opportunity for identification of small molecule agonists or antagonists of BACH2 function using high-throughput screening. Such assays may enable identification of novel therapeutic compounds to either augment or inhibit the suppressive function of BACH2 in immune activation.

## Methods

### Plasmids and generation of inducible-BACH2 and control reporter cell-based lines

A DNA sequence located at the putative *Ifng* + 18k enhancer containing a TRE element (5′–CAAAGAGGATGCCCCG**TGAGTCA**CTTACAAACCACAGC–3′) was concatenated three times and subcloned into the hygromycin-resistant luciferase reporter vector pNL2.2 (N107, Promega) upstream of a minimal promoter sequence (minP) and a cDNA sequence encoding luciferase. A human *BACH2* cDNA sequence was sub-cloned into the multiple cloning site (MCS) of the zeocin-resistant tetracycline inducible vector pcDNA.4/TO (V102020, Invitrogen) to generate the pcDNA4/BACH2-inducible vector. Together with pNL2.2 luciferase reporter, both plasmids were co-transfected into blasticidin-resistant Jurkat TRex cells (pcDNA6/TR) using the Amaxa Cell Line Nucleofector Kit V (VCA-1003, Lonza) following the manufacturer’s instructions. Stably transfected cells were selected by culturing cells in the presence of 100 μg/ml hygromycin (10687010, Invitrogen) and 200 μg/ml zeocin (R25001, Invitrogen) for 2 weeks. For the control line, only the hygromycin resistant-pNL2.2 luciferase reporter was transfected and cells were treated with hygromycin alone. Single cell clones were established by limiting dilution. Cells were cultured in RPMI medium (11875085, Gibco) containing 10% tetracycline free fetal bovine serum (P30-3602, PAN Biotech), 50,000 Units of penicillin–streptomycin (15140122, Gibco), 0.1 X glutamax (35050061, Gibco), 0.25 μg/ml of amphotericin B (15290026, Gibco) and 10 μg/ml of blasticidin (R21001, Invitrogen) and maintained after selection in half the concentration of the indicated selection antibiotics at 37 °C with 5% CO_2_.

### Sanger sequencing and data analysis

Inserts regions of the constructed vectors pNL2.2 *Ifng* + 18 k reporter and pcDNA.4/BACH2-inducible were confirmed using Sanger sequencing. Primers were designed for sequencing of pNL2.2 *Ifng* + 18k reporter vector as follows: Fw: ‘5–TCGATAGTACTAACATACGC–3’ and Rv: ‘5–GTTGTAGCCGGCTGTCTGTCG–3’. A primer walk strategy was followed to verify the pcDNA.4/BACH2-inducible vector insert and involved designing five different forward and reverse primer sequences as follows: Fw1: ‘5–CGCAAATGGGCGGTAGGCGTG–3’; Fw2: ‘5–ACGATGGATTCAGAGACGGC–3’; Fw3: ‘5–CTTAAGGTCTCTGTTCAGC–3’; Fw4: ‘5–AATCAAAGTCTGCCCTCG–3’; Fw5: ‘5–AATTTAGAATGTGAAATCCG–3’; and Rv1: ‘5–TAGAAGGCACATCGAGG–3’; Rv2: ‘5–TTTCTCACACACCAATTTGC–3’; Rv3: ‘5–GAATAGGAAGAGCAGGAGC–3’; Rv4: ‘5–TCCACACTTTTCGTTATGC–3’; Rv5: ‘5–TCATCCTCCTCCTCTCCTGC–3’. Sequencing data were analysed using FinchTv 1.4.0 software (Geospiza) and ChromasPro 2.1.9 software (Technelysium) for pNL2.2 *Ifng* + 18k reporter and pcDNA4/BACH2 inducible-vector respectively. Images of the confirmed insert sequences were merged after data analysis with Adobe Photoshop CS6 software (Adobe Creative Suite 6 Master Collection).

### Luciferase assay

Clonally derived cell lines were treated with or without tetracycline (T8032, Sigma-Aldrich) for 18 h. Subsequently, cells were stimulated with phorbol 12-myristate 13-acetate (PMA) (P1585, Sigma-Aldrich) and ionomycin (I0634, Sigma-Aldrich) at 25 ng/ml and 1.25 μg/ml respectively, if not otherwise stated, for 6 h in replenished culture medium containing tetracycline. Luciferase expression was acquired using the Nano-Glo Luciferase Assay System kit (N1130, Promega) following the manufacturer’s instructions. Luciferase signal was measured using a PHERAstar FS spectrophotometer. Data were analysed using GraphPad Prism 8 software.

### Western blotting

Selected clones were treated with or without tetracycline for 18 h. The cells were harvested and washed twice in phosphate-buffered saline (PBS). Cells were lysed in RIPA buffer (89901, Thermo Scientific) containing protease inhibitors (11836170001, Sigma-Aldrich). Total protein concentration was quantified using BCA assay (23225, Thermo Scientific) and normalised protein amount was loaded on SDS-PAGE gels followed by semi-dry western blotting. BACH2 protein was detected using BACH2-specific antibody (D3T3G Rabbit mAb, 80775S, Cell Signalling Technology). Detection of Jun family members was conducted with primary anti-c-Jun antibody (N, clone sc-45, J1713, Santa Cruz Biotechnology), anti-JunB antibody (210, clone sc-73, J1813, Santa Cruz Biotechnology) and anti-JunD antibody (329, clone sc-74, A3113, Santa Cruz Biotechnology). As a loading control β-actin protein was stained using anti-β-actin antibody (clone AC-74, A5316, Sigma-Aldrich). The specificity of anti-BACH2 antibody reactivity was tested by adding a BACH2 specific blocking peptide (38475S, Cell Signalling Technology) during primary antibody staining. Stripping of primary and secondary antibodies was performed by incubating the membrane in Restore Western Blot Stripping Buffer (21059, Thermo Scientific) followed by re-probing as described above. Protein quantification was conducted using ImageJ software^25^.

### RGFP966 inhibitor treatment

Inducible-BACH2 reporter cells were plated and pre-treated with or without 1 μg/ml tetracycline for 5 h prior to RGFP966 (16917, Cayman Chemical Company) inhibitor addition. The tetracycline pre-treated cells were additionally treated with 12.5 μM or without RGFP966 for 12 h following protein extraction or PMA/ionomycin stimulation for 6 h as described previously. Subsequently, BACH2 protein level detection with western blotting or luciferase activity measurements were performed using methods outlined above.

### Imaging

Cells from the inducible-BACH2 reporter line were pre-treated with titrated concentrations of tetracycline (namely 0.0024 μg/ml, 0.012 μg/ml, 0.5 μg/ml and 1 μg/ml) or without for 18 h. Stimulation of cells with PMA/ionomycin at above concentrations followed for 5 h. Cells were imaged prior and subsequently to luciferase substrate (Nano-Glo Live Cell Assay System kit (N2011, Promega)) addition following the manufacturer’s instructions. Luminescence and brightfield images were captured using a Nikon Ti-E microscope, Andor iXon Ultra EM-CCD camera, Nikon 20 × 0.8 NA objective, OKO lab environment chamber at 36 °C with 5% CO2 and Nikon Elements with JOBS module software. A 3 × 3 montage of images was acquired in each well with the camera set to maximum sensitivity (300 EM gain, 5.1 amplifier gain) using 10 s and 50 ms exposure times for luminescence and brightfield channels respectively. Images were processed and quantified with FIJI^26^ using the PureDenoise plug-in^27^ to improve the background of the luminescence images and the StarDist plug-in^28^ to create cell segmentation masks.

### Statistical analysis

Statistical tests of luciferase assays were performed using unpaired two-tailed Student’s *t* tests and two-way ANOVA with Bonferroni multiple comparisons correction where specified. All the luciferase measurements were conducted with at least three technical replicates per condition.

## Supplementary information

Supplementary Information.

## References

[1] Josefowicz SZ, Lu LF, Rudensky AY (2012). Regulatory T cells: mechanisms of differentiation and function. Annu Rev Immunol.

[2] Sakaguchi S, Yamaguchi T, Nomura T, Ono M (2008). Regulatory T cells and immune tolerance. Cell.

[3] Benoist C, Mathis D (2012). Treg cells, life history, and diversity. Cold Spring Harb Perspect Biol.

[4] Vignali DA, Collison LW, Workman CJ (2008). How regulatory T cells work. Nat Rev Immunol.

[5] Quezada SA, Peggs KS, Simpson TR, Allison JP (2011). Shifting the equilibrium in cancer immunoediting: from tumor tolerance to eradication. Immunol Rev.

[6] Stockis J, Roychoudhuri R, Halim TYF (2019). Regulation of regulatory T cells in cancer. Immunology.

[7] Henning AN, Roychoudhuri R, Restifo NP (2018). Epigenetic control of CD8(+) T cell differentiation. Nat Rev Immunol.

[8] Reinke AW, Baek J, Ashenberg O, Keating AE (2013). Networks of bZIP protein-protein interactions diversified over a billion years of evolution. Science.

[9] Turner R, Tjian R (1989). Leucine repeats and an adjacent DNA binding domain mediate the formation of functional cFos-cJun heterodimers. Science.

[10] Glover JN, Harrison SC (1995). Crystal structure of the heterodimeric bZIP transcription factor c-Fos-c-Jun bound to DNA. Nature.

[11] Roychoudhuri R (2016). BACH2 regulates CD8(+) T cell differentiation by controlling access of AP-1 factors to enhancers. Nat Immunol.

[12] Igarashi K, Kurosaki T, Roychoudhuri R (2017). BACH transcription factors in innate and adaptive immunity. Nat Rev Immunol.

[13] Roychoudhuri R (2013). BACH2 represses effector programs to stabilize T_reg_-mediated immune homeostasis. Nature.

[14] Oyake T (1996). Bach proteins belong to a novel family of BTB-basic leucine zipper transcription factors that interact with MafK and regulate transcription through the NF-E2 site. Mol Cell Biol.

[15] Kuwahara M (2016). Bach2-Batf interactions control Th2-type immune response by regulating the IL-4 amplification loop. Nat Commun.

[16] Ivashkiv LB (2018). IFNgamma: signalling, epigenetics and roles in immunity, metabolism, disease and cancer immunotherapy. Nat Rev Immunol.

[17] Roychoudhuri R, Eil RL, Restifo NP (2015). The interplay of effector and regulatory T cells in cancer. Curr Opin Immunol.

[18] Tanaka H (2016). Epigenetic regulation of the Blimp-1 gene (Prdm1) in B cells involves Bach2 and histone deacetylase 3. J Biol Chem.

[19] Malvaez M (2013). HDAC3-selective inhibitor enhances extinction of cocaine-seeking behavior in a persistent manner. Proc Natl Acad Sci U S A.

[20] Grant FM (2020). BACH2 drives quiescence and maintenance of resting Treg cells to promote homeostasis and cancer immunosuppression. J Exp Med.

[21] Sidwell T (2020). Attenuation of TCR-induced transcription by Bach2 controls regulatory T cell differentiation and homeostasis. Nat Commun.

[22] Ying C (2015). Enhancement of regulatory T cell-like suppressive function in MT-2 by long-term and low-dose exposure to asbestos. Toxicology.

[23] Watson PJ, Fairall L, Santos GM, Schwabe JW (2012). Structure of HDAC3 bound to co-repressor and inositol tetraphosphate. Nature.

[24] Li J (2000). Both corepressor proteins SMRT and N-CoR exist in large protein complexes containing HDAC3. EMBO J.

[25] Schneider CA, Rasband WS, Eliceiri KW (2012). NIH Image to ImageJ: 25 years of image analysis. Nat Methods.

[26] Schindelin J (2012). Fiji: an open-source platform for biological-image analysis. Nat Methods.

[27] Luisier F, Vonesch C, Blu T, Unser M (2010). Fast interscale wavelet denoising of Poisson-corrupted images. Signal Process.

[28] Schmidt, U., Weigert, M., Broaddus, C. & Myers, G. Cell detection with star-convex polygons, in *International Conference on Medical Image Computing and Computer-Assisted Intervention (MICCAI)* (Granada, Spain, 2018).

