# Supplementary material for: A cell-based bioluminescence assay reveals dose-dependent and contextual repression of AP-1-driven gene expression by BACH2
Source: Sci Rep. 2020 Nov 3;10:18902. doi: 10.1038/s41598-020-75732-z (PMC7641119; doi:10.1038/s41598-020-75732-z)

Supplemental Material

A cell-based bioluminescence assay reveals dose-dependent and contextual repression of AP-1-driven gene expression by BACH2

Panagiota Vardaka[1,2], Teresa Lozano[2], Christopher Bot[3], Jonathan Ellery[3], Sarah K Whiteside[1,2], Charlotte J Imianowski[1,2], Stuart Farrow[3], Simon Walker[4], Hanneke Okkenhaug[4], Jie Yang[1,2], Klaus Okkenhaug[1], Paula Kuo[1,2] and Rahul Roychoudhuri[1,2]

[1] Department of Pathology, University of Cambridge, Tennis Court Road, CB2 1QP, UK.

[2] Laboratory of Lymphocyte Signalling and Development, Babraham Institute, Cambridge, CB22 3AT, UK.

[3] CRUK Therapeutic Discovery Laboratories, Babraham Research Campus, CB22 3AT, UK.

[4] Imaging Facility, Babraham Institute, Cambridge, CB22 3AT, UK.

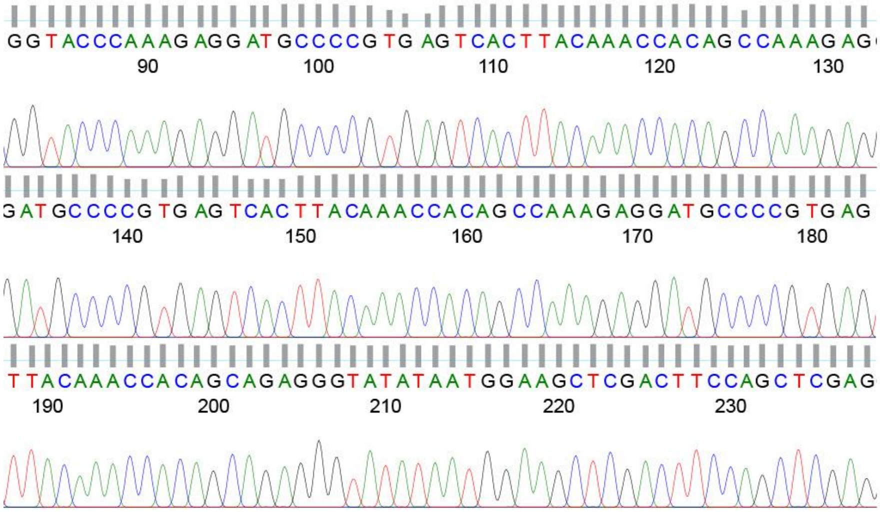

Supplementary Figure 1. Sanger sequencing of insert region from pNL2.2 *Ifng* +18k reporter vector. Chromatogram of the pNL2.2 *Ifng* +18k reporter vector insert containing a 3x concatenated sequence with a TPA-response element (TRE) embedded motif from the *Ifng* +18k enhancer region.

a

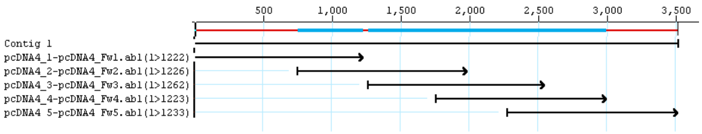

b

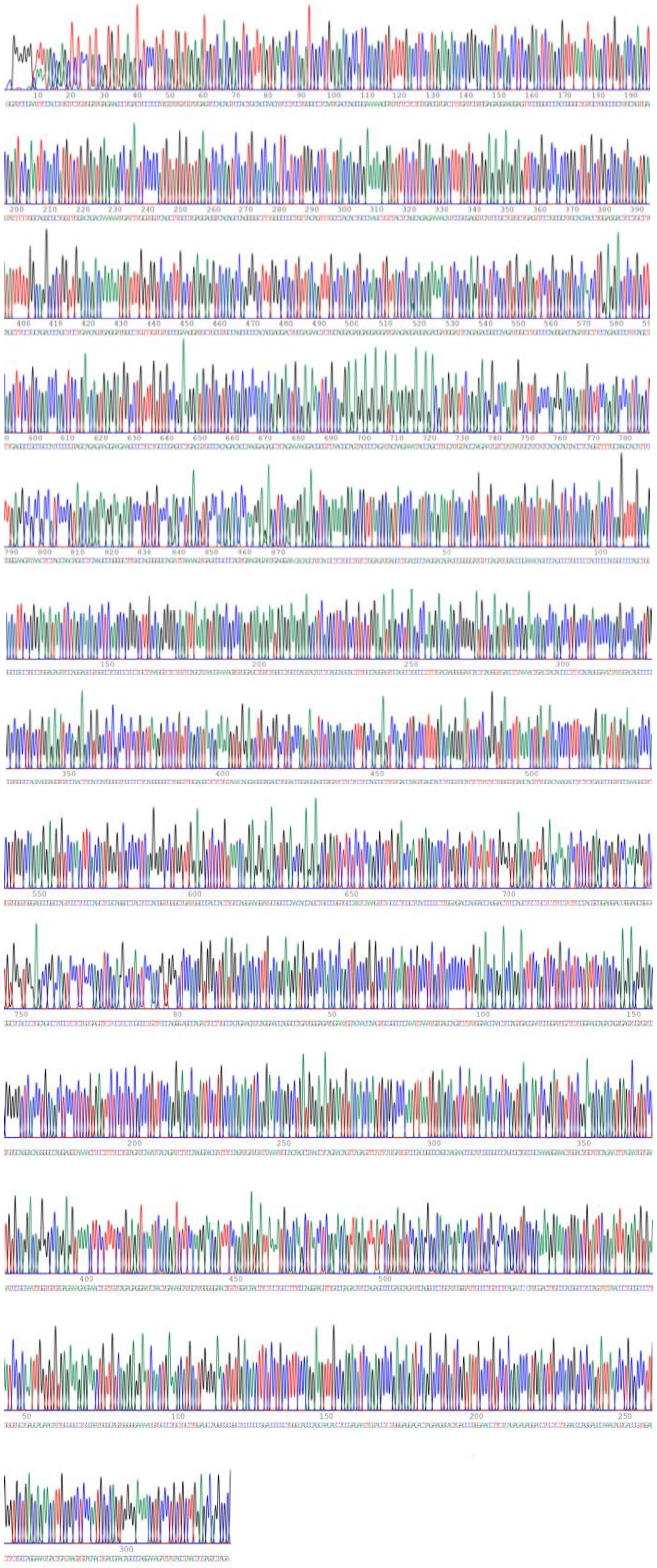

Supplementary Figure 2. Sanger sequencing of insert region of pcDNA4-BACH2 inducible vector. a, and **b,** Sequence assembly of human *BACH2* cDNA subcloned into pcDNA4 vector data. **a,** Representation of primer walk strategy followed for insert sequencing. **b,** Chromatogram showing pcDNA4-BACH2 inducible vector insert sequence after data analysis.

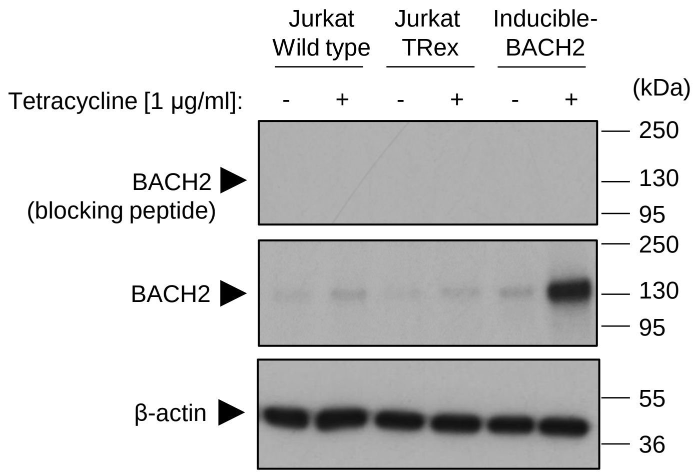

Supplementary Figure 3. Specific induction of BACH2 protein expression upon tetracycline treatment of the BACH2 reporter line. Western blot of total protein lysates with or without tetracycline pre-treatment. The specificity of the BACH2 signal was confirmed by pre-incubating the primary anti-BACH2 antibody with an anti-BACH2 blocking peptide prior to primary antibody staining.

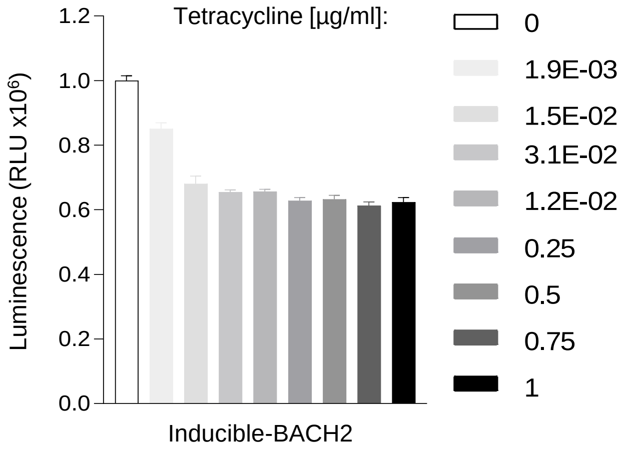

Supplementary Figure 4. Signal-driven luciferase expression of inducible-BACH2 reporter cells pre-treated with titrated tetracycline doses. Signal-driven luminescence of inducible-BACH2 reporter cells at given tetracycline concentrations following stimulation with PMA/ionomycin showing minimal luminescence at 1 µg/ml tetracycline. Data show 4 culture replicates per condition. Bars and error represent mean (SD).

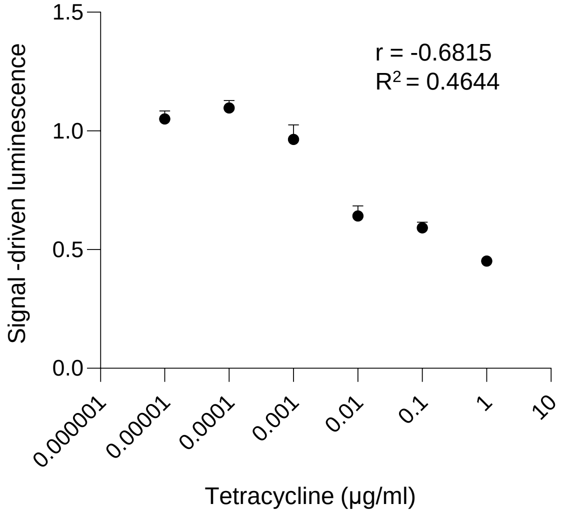

Supplementary Figure 5. Association signal-driven luciferase expression and tetracycline dose in the BACH2 reporter cell line. Signal-driven luminescence of inducible-BACH2 reporter cells at given tetracycline concentrations following stimulation with PMA/ionomycin. Data are representative of 2 independently repeated experiments with 3 culture replicates per condition. Bars and error represent mean (SD).

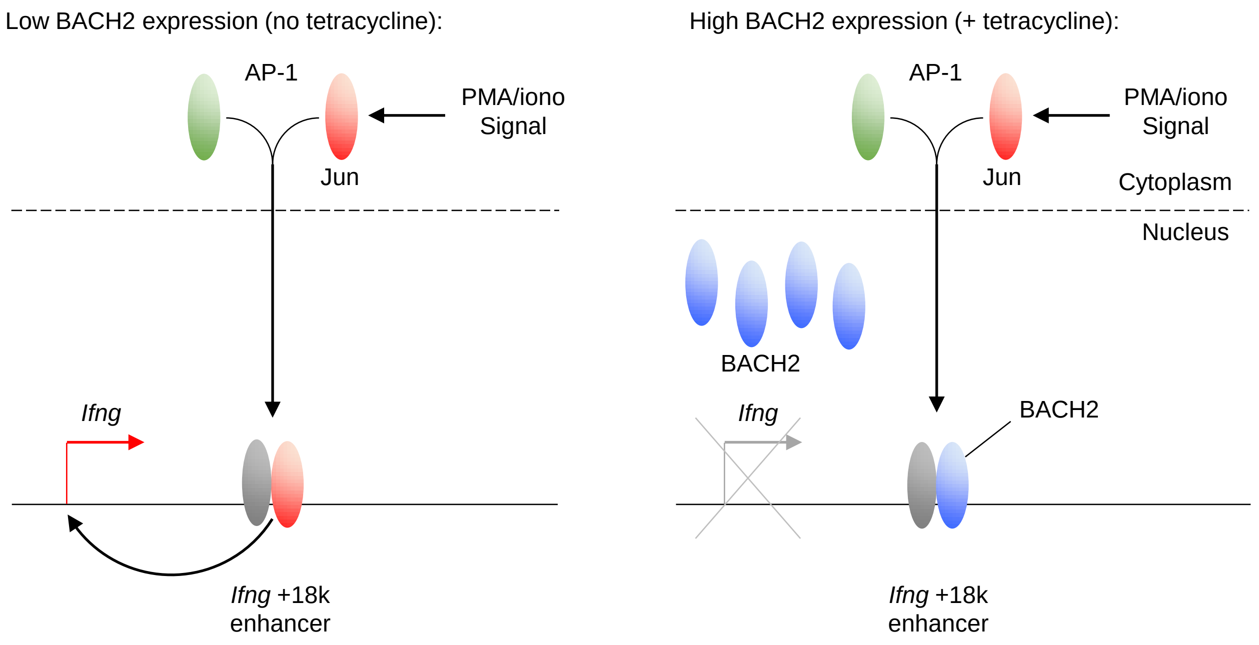

Supplementary Figure 6. Schematic representation of the proposed function of BACH2 in the developed reporter assay.

Additional Figures:

Full image from Fig. 2b:

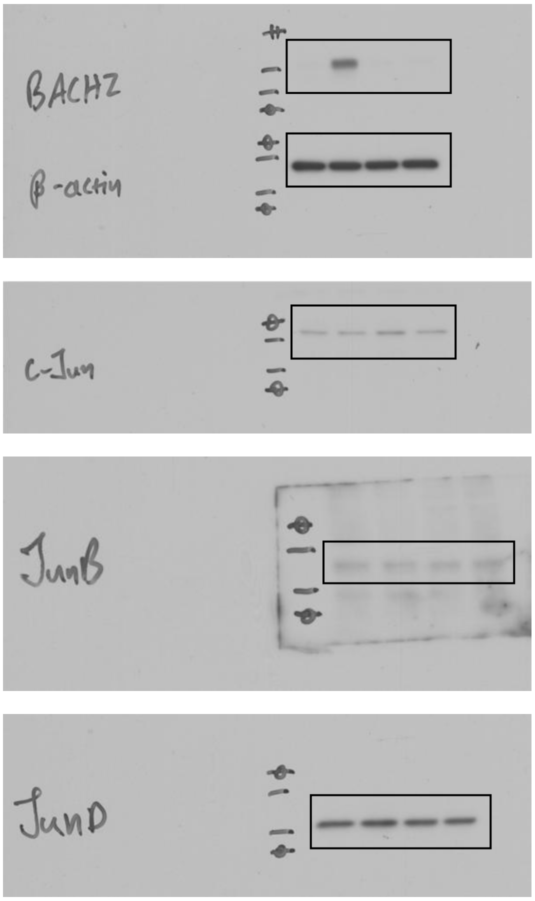

Additional Figures:

Full image from Fig. 3b:

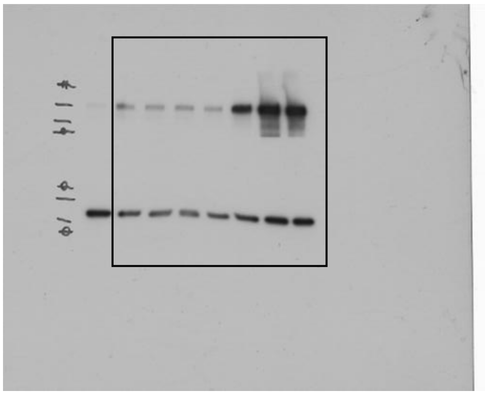

Additional Figures:

Full image from Fig. 6c:

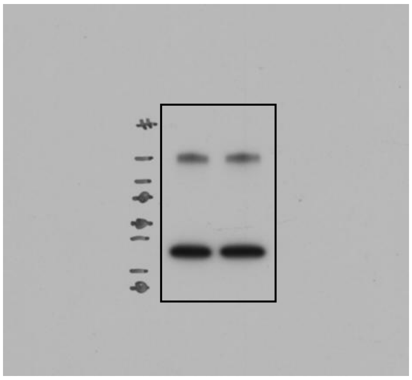

Additional Figures:

Full image from Supplementary Figure 3:

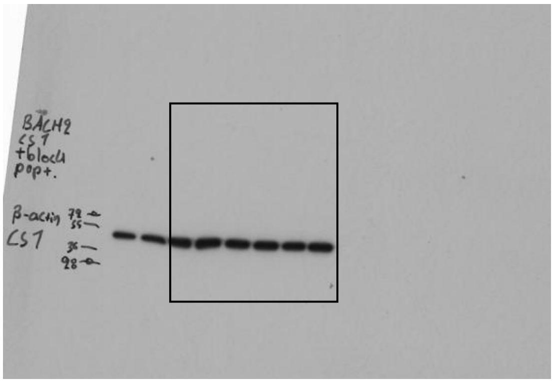

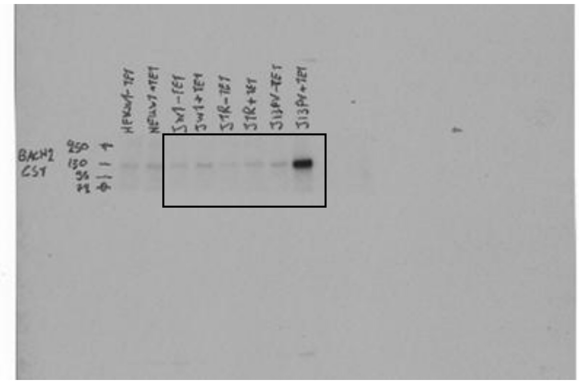

Supplement: Supplementary file 1 — Supplementary Information. [file 41598_2020_75732_MOESM1_ESM.pdf]
